# Supplementary material for: Causal Relationship between Aging and Anorexia Nervosa: A White-Matter-Microstructure-Mediated Mendelian Randomization Analysis
Source: Biomedicines. 2024 Aug 16;12(8):1874. doi: 10.3390/biomedicines12081874 (PMC11351342; doi:10.3390/biomedicines12081874)
Supplement: Supplementary file 1 [file biomedicines-12-01874-s001.zip › Supplementary Figure/Figure S4/Singe SNP plot of MR analysis for TL to ubm-b-1609.pdf]

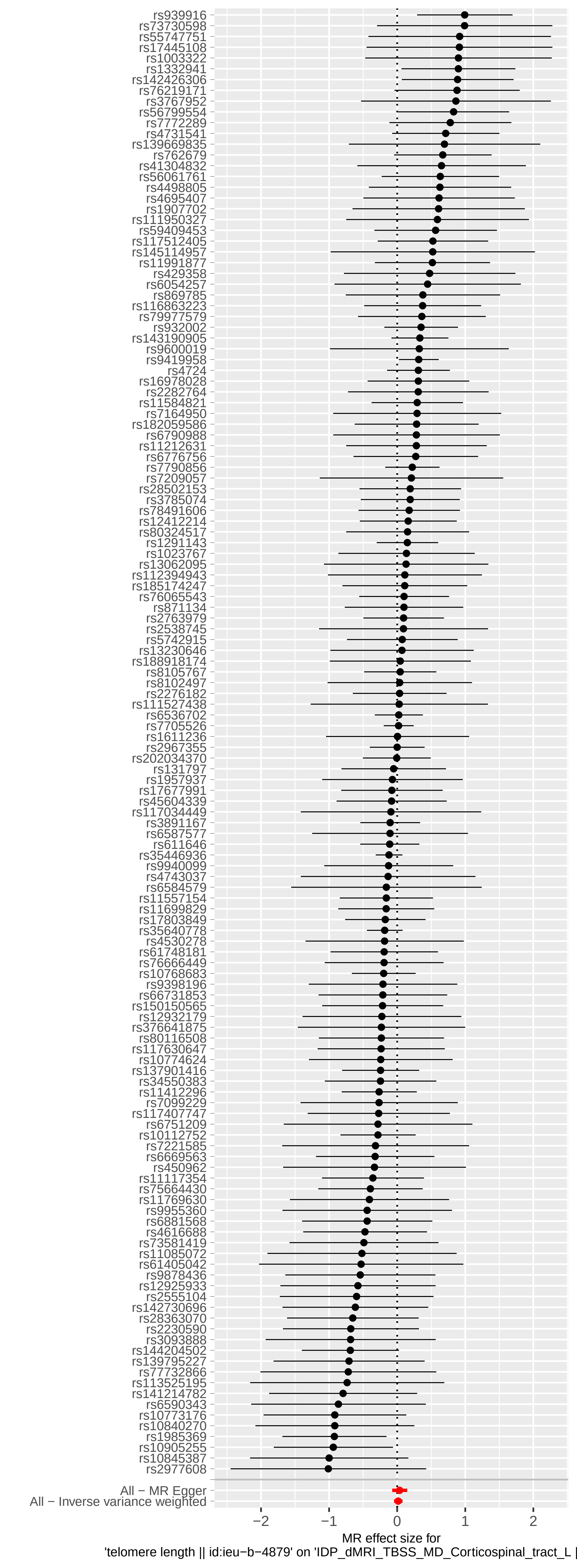
rs939916
rs73730598
rs55747751
rs17445108
rs1003322
rs1332941
rs142426306
rs76219171
rs3767952
rs56799554
rs7772289
rs4731541
rs139669835
rs762679
rs41304832
rs56061761
rs4498805
rs4695407
rs1907702
rs111950327
rs59409453
rs117512405
rs145114957
rs11991877
rs429358
rs6054257
rs869785
rs116863223
rs79977579
rs932002
rs143190905
rs9600019
rs9419958
rs4724
rs16978028
rs2282764
rs11584821
rs7164950
rs182059586
rs6790988
rs11212631
rs6776756
rs7790856
rs7209057
rs28502153
rs3785074
rs78491606
rs12412214
rs80324517
rs1291143
rs1023767
rs13062095
rs112394943
rs185174247
rs76065543
rs871134
rs2763979
rs2538745
rs5742915
rs13230646
rs188918174
rs8105767
rs8102497
rs2276182
rs111527438
rs6536702
rs7705526
rs1611236
rs2967355
rs202034370
rs131797
rs1957937
rs17677991
rs45604339
rs117034449
rs3891167
rs6587577
rs611646
rs35446936
rs9940099
rs4743037
rs6584579
rs11557154
rs11699829
rs17803849
rs35640778
rs4530278
rs61748181
rs76666449
rs10768683
rs9398196
rs66731853
rs150150565
rs12932179
rs376641875
rs80116508
rs117630647
rs10774624
rs137901416
rs34550383
rs11412296
rs7099229
rs117407747
rs6751209
rs10112752
rs7221585
rs6669563
rs450962
rs11117354
rs75664430
rs11769630
rs9955360
rs6881568
rs4616688
rs73581419
rs11085072
rs61405042
rs9878436
rs12925933
rs2555104
rs142730696
rs28363070
rs2230590
rs3093888
rs144204502
rs139795227
rs77732866
rs113525195
rs141214782
rs6590343
rs10773176
rs10840270
rs1985369
rs10905255
rs10845387
rs2977608
All − MR Egger
All − Inverse variance weighted
−2
−1
0
1
2
MR effect size for
'telomere length || id:ieu−b−4879' on 'IDP_dMRI_TBSS_MD_Corticospinal_tract_L |
